# CircEPDR1 regulates proliferation and differentiation of goat skeletal muscle satellite cells through miR-345-3p/Akirin1 axis

**DOI:** 10.5713/ab.24.0845

**Published:** 2025-03-31

**Authors:** Song Shi, Diansong Duan, Jing Luo, Zihao Zhao, Tao Ma, Dinghui Dai, Siyuan Zhan, Jiaxue Cao, Jiazhong Guo, Tao Zhong, Linjie Wang, Hongping Zhang, Li Li

**Affiliations:** 1 Farm Animal Genetic Resources Exploration and Innovation Key Laboratory of Si-chuan Province, Sichuan Agricultural University, Chengdu, China

**Keywords:** Akirin1, CircEPDR1, Goat, MiR-345-5p, Skeletal Muscle

## Abstract

**Objective:**

Circular RNAs (circRNAs) play pivotal roles in regulating the growth and development of mammalian skeletal muscle This study aims to provide new insights into the molecular regulatory network that underlies skeletal muscle development, presenting valuable targets to enhance and optimize livestock production performance.

**Methods:**

In this study, we anchored a novel circRNA from previous circRNA sequencing data from goat skeletal muscle and named it circEPDR1, which was confirmed through en-zymatic digestion and Sanger sequencing. Functional investigation of circEPDR1 included overexpression experiments in goat muscle satellite cells (MuSCs), and its sub-cellular localization was determined using nuclear-cytoplasmic separation. Bioinformatics analysis was employed to reveal a circRNA-miRNA-RNA regulatory pathway involving circEPDR1. This process was further validated through Dual-luciferase reporter assay, real-time fluorescence quantitative polymerase chain reaction, Western blotting, EdU incorporation, CCK-8 cell viability tests, and immunofluorescence experiments.

**Results:**

We newly identified circEPDR1, which is produced by reverse splicing the second and third exons of the EPDR1 gene. circEPDR1 was enriched in skeletal muscle and dynamically expressed during the growth of goat MuSCs. In addition, circEPDR1 significantly inhibited the proliferation while promoting myogenic differentiation of goat MuSCs. The dominant cytoplasm distribution and enrichment by the AGO2 protein imply that circEPDR1 potentially works as a competitive endogenous RNA. Mechanically, miR-345-3p directly targeted circEPDR1, and its function in myogenesis was completely reversed to that of circEPDR1. Meanwhile, miR-345-3p negatively regulated Akirin1 mRNA through their base-pairing. Similar to circEPDR1, Akirin1 suppressed proliferation while also promoting differentiation.

**Conclusion:**

This study demonstrates that circEPDR1 controls a vital role in regulating the proliferation and differentiation of goat MuSCs through the circEPDR1/miR-345-3p/Akirin1 axis.

## INTRODUCTION

Skeletal muscle, one of the most prevalent tissue types in mammals, accounts for 30% to 40% of body weight and plays a crucial role in vital life activities including locomotion, immune function, and energy metabolism [1,2]. Its development is closely related to livestock growth and meat production capabilities [3]. The formation of skeletal muscle is a complex-process that initiates with the activation of muscle satellite cells (MuSCs). Subsequently, these cells proliferate and differentiate into myoblasts, which fuse to create multinucleated myotubes that eventually mature into myofibers. These myofibers densely pack to form skeletal muscle tissue [4]. This transition from stem cells to mature myofibers is carefully orchestrated by several gene families, such as paired box proteins, myogenic regulatory factors, and myocyte enhancer factors (MEFs) [5,6]. Notably, the abundant and diverse non-coding RNAs (ncRNAs) in skeletal muscle tissues play a pivotal role in the complex regulation of mammalian skeletal muscle growth and development [7]. For example, microRNAs (miRNAs) can either promote or inhibit cell differentiation by targeting specific transcription factors and signaling pathways [8]. Meanwhile, long non-coding RNAs (lncRNAs) promote cell proliferation and differentiation by mediating epigenetic [9], transcriptional, and post-transcriptional regulation [10].

Besides miRNA and lncRNAs, emerging circRNAs have been a hotspot for uncovering novel mechanisms underlying skeletal muscle development. This is due to their unique covalent closed-loop structures and diverse biological functions [11]. Despite being relatively low in abundance, circRNAs exhibit high evolutionary conservation and are widely expressed across species [12]. They also display remarkable tissue and cell specificity [13]. circRNAs finely regulate skeletal muscle development through multiple mechanisms, including interactions with specific proteins. In addition, some circRNAs are translated into small peptides, such as circNEB-derived peptide significantly promotes the proliferation and differentiation of bovine adult myoblasts [14]. Notably, serving as competitive endogenous RNA (ceRNA) is one of the most common and important functions for cytoplasm circRNAs [15]. Through their abundant miRNA binding sites, circRNAs indirectly regulate the mRNAs of targeted myogenic genes by effectively competing with their shared miRNAs [16]. This provides a favorable molecular environment for muscle growth, differentiation, and repair. For example, circfut10 [17], circMYBPC1 [18], and circNDST [19], significantly affect the proliferation and differentiation of livestock skeletal muscle stem cells and adult myoblasts. These findings have greatly expanded our understanding of the multilevel and multidimensional roles of circRNAs in skeletal muscle development.

Although the role of circular RNAs in skeletal muscle development has garnered increasing attention, our full understanding of their specific mechanisms remains to be extensively unveiled. Drawing upon our previous circRNA sequencing data, we successfully identified a novel circEPDR1, and in the subsequent in-depth investigation of its function and mechanism of action, we have clarified that circEPDR1 plays a crucial role in the development and regeneration of skeletal muscle in goats. This study provides a scientific foundation for extensively exposing the mechanisms underpinning mammal skeletal muscle development.

## MATERIALS AND METHODS

### Ethics statement

All procedures in this study strictly followed the Regulations for the Administration of Experimental Animals issued by the Ministry of Science and Technology of China (Chengdu, China). Furthermore, all protocols and the use of animals were thoroughly reviewed and approved by the Institutional Animal Care and Use Committee of Sichuan Agricultural University (permit no. Dky-2022202056).

### Animals, samples collection, and muscle satellite cells isolation

Four key developmental stages (early embryonic stage E45, mid-late embryonic stage E105, postnatal stage B3, and B150) for goat muscle development were selected. Nine tissues from humanly sacrificed Jianzhou Da’er goats (n = 3 at each stage) were rapidly sampled, including the longissimus dorsi muscle (LD), semitendinosus muscle (ST), psoas major muscle (PM), gastrocnemius muscle (GA), heart, liver, spleen, lung, and kidney. These tissues were immediately stored in liquid nitrogen for further investigation.

Primary MuSCs were isolated and cultured from the LD muscle of B3 goats by enzymatic digestion [20]. Briefly, cells were purified by Percoll gradient centrifugation and identified by MyHC and Pax7 immunofluorescence staining. The isolated cells were packed in cryogenic storage tubes and stored in liquid nitrogen for future use.

### Cell culture and transfection

MuSCs were initially seeded into 6-well plates (2×10^5^ cells per well) or 12-well plates (1×10^5^ cells per well) and cultivated in a proliferation medium (GM). This GM was composed of high-glucose Dulbecco’s modified Eagle’s Medium (DMEM) fortified with 10% fetal bovine serum (Gibco, Grand Island, NE, USA) and 2% Penicillin & Streptomycin (Invitrogen, Carlsbad, CA, USA). Once the cells achieved 80% to 90% confluence, they were induced to differentiate by transferring to a DMEM differentiation medium containing 2% horse serum (Gibco) and 1% Penicillin & Streptomycin. This differentiation process continued in a 37°C incubator with a 5% CO_2_.

For functional studies, overexpression plasmids were introduced into the cells utilizing Lipofectamine 3000 (Thermo Fisher Scientific, Waltham, MA, USA). Subsequently, the transfected cells were collected at two distinct time points: 48 h post-transfection for RNA and 72h for protein detection.

### Extraction of RNA, gDNA, and real-time fluorescence quantitative polymerase chain reaction

Following the manufacturer’s guidelines, total RNA was extracted from cell or tissue samples employing the TRIzol reagent. The extracted RNA was then subjected to reverse transcription into cDNA and miRNA, utilizing the HiScript QRT SuperMix for qPCR (+gDNA wiper) and the Mir-X miRNA First-Strand Synthesis Kit, respectively. Additionally, reverse transcription of RNA containing Poly(A) tails was performed using PrimeScript RT reagent Kit (RR037A; Takara, Beijing China).

To extract gDNA from goat LD, the sample was first grounded, and added anhydrous ethanol to precipitate the DNA. After forming DNA clumps by resting, the precipitates were collected with a sterile centrifuge tube. After washing with 70% ethanol 2 to 3 times and drying, the precipitation was dissolved in deionized water to obtain a high-purity gDNA solution. Finally, the solution was packaged and stored in a −20°C refrigerator.

Real-time quantitative polymerase chain reaction (RT-qPCR) was executed on the CFX96 Touch Real-Time PCR Detection System (Bio-Rad, Hercules, CA, USA). For normalization purposes, mouse glyceraldehyde-3-phosphate dehydrogenase (GAPDH) and U6 small nuclear RNA (U6) were employed as endogenous controls for mRNA and miRNA, respectively. Relative fold changes in the expression of candidate genes were determined using the 2^−ΔΔCT^ method.

### Primer design and vector construction

Primers were designed using the Primer Premier V5.0 software and their specificity was confirmed by the blast tool available on National Center for Biotechnology Information (https://www.ncbi.nlm.nih.gov/). Qinke Biological Co., Ltd. carried out the subsequent primer synthesis. The primers used here are detailed in Supplement 1.

For overexpression, PCD5-ciR and pEGFP-N1 sourced from GeneSeed Biotech (Guangzhou, China) were selected to construct vectors for circEPDR1 and Akirin1, respectively. Furthermore, psiCHECK2 provided by Qinke Biological Co., Ltd. PCR was chosen as the dual-luciferase reporter vector combined with Sanger sequencing to ensure the accuracy of all these vectors constructed.

### RNase R treatment

After adding 10 U of RNase R (R0301; Geneseed Biotech) to 2.5 μg of total RNA and allowing the mixture to incubate at 37°C for 15 minutes, electrophoresis was employed to detect the presence of circEPDR1 and EPDR1.

### Western blotting and antibodies

Initially, total protein was extracted from cells or tissues, and its quantity was determined using the Bicinchoninic Acid Assay Protein Assay Kit (Bestbio, Shanghai, China). The extracted proteins were then resolved through sodium dodecyl sulfate polyacrylamide gel electrophoresis electrophoresis and transferred onto a polyvinylidene fluoride membrane. The membrane was blocked with 5% skim milk dissolved in Tris-buffered saline containing Tween 20 to prevent non-specific binding. Subsequently, the membrane was incubated sequentially with primary and secondary antibodies, and the proteins were detected using an Enhanced Chemiluminescence detection kit (Beyotime, Shanghai, China). The results were observed and captured using a gel imaging system (e-BLOT Touch Imager pro; e-BLOT, Shanghai, China), and a semi-quantitative analysis was performed with Image J V1.54 to normalize the target protein levels against β-tubulin.

The primary es utilized here included Pax7 (1:200, sc-81975; Santa Cruz, Dallas, TX, USA), PCNA (1:500, GB12010-100; Servicebio, Wuhan, China), MyoD (1:500, YK0160; Immunoway, Beijing, China), MyHC (1:200, sc-376157 Santa Cruz), and β-tubulin (1:5000, 250007; ZENBIO, Chengdu, China). The secondary antibodies employed were anti-mouse IgG (1:5000, AS003; ABclonal, Wuhan, China) and anti-rabbit IgG (1:5000, AS014; ABclonal).

### Cells number analysis

In the CCK-8 assay, cells with a density ranging from 1×10^4^ to 1×10^5^ were seeded into 96-well plates. Once the cells attained 40% confluence, they were transfected with plasmids. At 6, 24 and 48 h, the cells were incubated with Cell Counting Kit-8 reagent (Servicebio) for durations of 2 h, respectively. The absorbance of the CCK-8-treated cells was measured at 450 nm.

Regarding EdU detection, cells were cultured in 6-well plates until they achieved 50% confluence. Subsequently, they were transiently transfected with plasmids. After allowing for 48 h of proliferation, EdU testing was conducted using the EdU kit’s guidelines (Click-iT EdU-555; Servicebio). Briefly, Cells in the s-phase were stained with EdU (red), and their nuclei were stained with Hoechst 33342 (blue) (magnification, ×100 μm). The EdU-positive (EdU^+^) cells were visualized under a fluorescent microscope (Olympus, Olympus Corporation, Tokyo, Japan), and ImageJ V1.54 was used to count EdU-positive cells. Finally, the percentage of EdU^+^ cells was calculated (EdU^+^ cells/total number of cells).

### Nuclear-cytoplasmic separation

Nuclear and cytoplasmic RNA purification was performed using the Cytoplasmic & Nuclear RNA Purification Kit (Norgen, Thorold, ON, Canada). Initially, cells were harvested and kept on ice. The cells underwent two washes with pre-cooled phosphate-buffered saline (PBS) or ultrapure H_2_O to eliminate cell debris and non-viable cells. Subsequently, the cells were digested with trypsin and centrifuged. And cell pellet was resuspended in lysis buffer and incubated on ice for 30 min to facilitate cell membrane disruption. Following incubation, the cell suspension was centrifuged at 3,000 rpm for 8 min, resulting in a pellet enriched with the nucleus and a supernatant containing predominantly cytoplasmic constituents. The supernatant was then transferred to a fresh tube and centrifuged once more to eliminate any residual nucleus further. Meanwhile, the nuclear pellet was resuspended in a buffer, and the centrifugation and washing steps were repeated to completely remove any lingering cytoplasmic components.

### Dual-luciferase reporter assay

The Dual-Luciferase Reporter Assay Kit (TransGen Biotech, Beijing, China) was used here. Firstly, reporter gene vectors (circEPDR1 W, circEPDR1 M, circEPDR1 W’, circEPDR1 M’ Akirin1 W, and Akirin1 M) containing the gene or sequence of interest, along with the Firefly Luciferase gene (F-Luc) and Renilla Luciferase gene (R-Luc), were constructed. Secondly, the recombinant plasmids were transfected into MuSCs at a density of 1×10^4^ to 1×10^5^ cells per well in a 96-well plate and cultured for 48 h, with plasmids carrying both Firefly and Renilla Luciferases being efficiently co-transfected. Subsequently, cells were collected and lysed to release the luciferases, and the corresponding substrates were added separately. Finally, a microplate reader (VarioSkan LUX, Thermo Fisher Scientific) was used to quantitatively measure the activities of the two luciferases, with the intensity of the emitted fluorescence serving as an indicator of the expression level of the target gene.

### RNA immunoprecipitation

RNA immunoprecipitation (RIP) experiments were performed utilizing the Pure Binding RIP Kit (Geneseed). Initially, cells were lysed to release their intracellular RNA-binding proteins. Subsequently, Argonaute2 (AGO2) antibodies were introduced to the cell lysates to selectively bind to the target RNA-binding proteins. Magnetic beads were then employed to precipitate the antibody-protein-RNA complexes, followed by rigorous washing to eliminate non-specific binding components. Afterward, the target RNA-binding proteins were dissociated from the antibodies using deionized water or a designated buffer. Finally, the immunoprecipitated RNAs were quantified using RT-qPCR to gain insights into their specific interactions.

### Immunofluorescence staining

When MuSCs achieved approximately 90% confluence, plasmid transfection was carried out. Following 2 to 4 days of differentiation induction, the cells were fixed with 4% paraformaldehyde for 20 min. Subsequently, permeabilization was enhanced by applying 0.5% Triton X-100 for 15 min, followed by blocking with 5% bovine serum albumin (SigmaAldrich, St. Louis, MO, USA) for 30 min to minimize non-specific antibody binding. The MyHC primary antibody (1:200, sc-376157; Santa Cruz) was then diluted at a ratio of 1:200 and incubated with the cells overnight. After washing with PBS, the cells were incubated with a goat anti-mouse IgG fluorescence secondary antibody diluted to 1:400. Nucleus (of the cell) were stained with 4′,6-diamidino-2-phenylindole. Finally, the expression patterns of MyHC were observed and analyzed under a fluorescent microscope (Olympus). ImageJ V1.54 was employed to count the myotube fusion rate (Number of multinucleated myotube nuclei/Total number of nuclei).

### Prediction of miRNA, target Gene, and translation potential of circEPDR1

In the process of miRNA prediction, we first use miRbase (https://www.mirbase.org/) to determine the sequence of miRNA. Subsequently, miRNA prediction results were obtained based on circRNA-seq data. These predicted results were integrated with miRNAs from authoritative databases such as miRDB (https://mirdb.org/), miRwalk (http://mirwalk.umm.uni-heidelberg.de/), miRmap (https://mirmap.ezlab.org), and TargetScan (https://www.targetscan.org/mmu_80/). Based on miRanda (https://www.bioinformatics.com.cn/) analysis, the complementation sequence and binding energy (ΔG, kCal/Mol) were determined. Similarly, in the prediction stage of target genes, we not only made full use of the resources of online prediction sites (https://www.omicstudio.cn/tool) but also integrated the prediction results of TargetScan. By finding the intersection of these resources, we targeted potential target genes. Subsequently, using Cytoscape V3.10.1 software, we constructed the protein-protein interaction (PPI) network of the circRNA-miRNA-Target gene.

To further explore the potential translation function of circRNA, we used an online website for exploring circRNA m^6^A modification site (http://www.cuilab.cn/sramp/), RNA binding protein (http://www.csbio.sjtu.edu.cn/bioinf/RBPsuite/) and open reading frame (http://www.ncbi.nlm.nih.gov/orffinder).

### Data analysis

To ensure the precision of experimental outcomes, at least three biological replicates were established for each treatment. The statistical analysis was conducted utilizing GraphPad Prism V8.0.2. The unpaired two-tailed t-test was used to compare two means. At the same time, one-way or two-way ANOVA (analysis of variance) was employed to analyze the mean difference among three or more independent samples once their standard deviation met Brown-Forsythe test and/or Bartlett’s test. Dun-can’s method was used for multiple comparisons. All data are presented as the mean±standard error of the mean. Statistical significance was ascertained based on a p-value threshold with *p<0.05, **p<0.01, and ^ns^p>0.05.

## RESULTS

### Identification and expression pattern of circEPDR1

**Figure 1:**
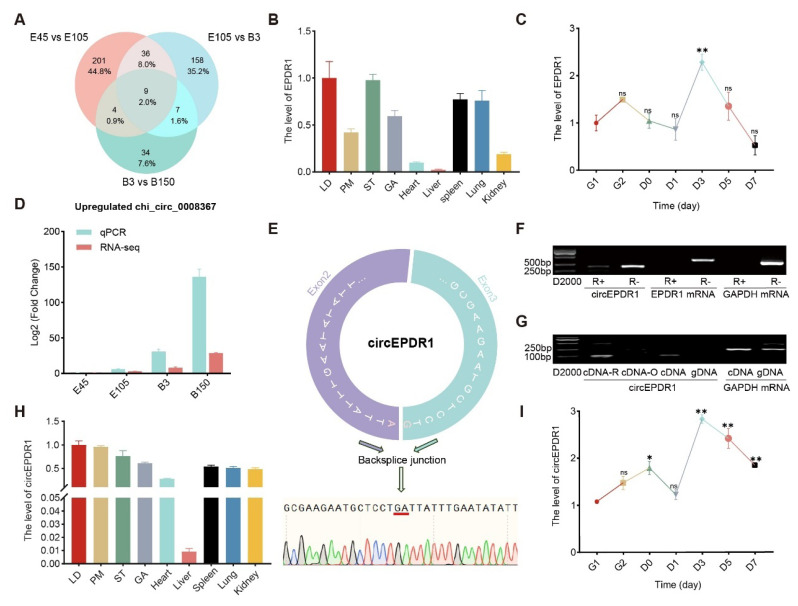
Profiles of circEPDR1 in goat tissues and MuSCs. (A) Venny map of circR-NA differentially expressed in LD muscle of goats at four stages. (B) The level of EPDR1 mRNA quantified using RT-qPCR in goat tissues. (C) EPDR1 mRNA detected by RT-qPCR in the proliferation and differentiation of goats MuSCs. (D) chi_circ_0008367 levels in goat LD evaluated by circular RNA sequencing and RT-qPCR. (E) The circular junction of circEPDR1 validated by Sanger sequencing. (F) CircEPDR1 levels detected in samples treated by RNase R. (G) CircEPDR1 amplified in gDNA and cDNA templates reversely transcripted using Random primers (cDNA-R), Oligo (dT) primers (cDNA-O), and common primer (cDNA). GAPDH works as a control. (H) Levels of circEPDR1 in goat tissues detected by RT-qPCR. (I) CircEPDR1 in the proliferation and myogenic differentiation of goats MuSCs quantified using RT-qPCR. Values are mean±SEM for three biological replicates, * p<0.05, ** p<0.01, and ^ns^ p>0.05. MuSC, muscle satellite cell; LD, longissimus dorsi muscle; RT-qPCR, real-time quantitative polymerase chain reaction; GAPDH, glyceraldehyde-3-phosphate de-hydrogenase; NS, not significant.

To anchor circRNAs closely related to skeletal muscle development, we analyzed circRNA sequencing data from the LD muscles of goats at four critical developmental stages (E45, E105, B3, and B150). A total of nine differentially expressed circRNAs were identified (Figure 1A). These circRNAs originate from 7 protein-coding genes, one uncharacterized ncRNA, and one unannotated region, respectively (Supplement 2). Using the online tool STRING (https://cn.string-db.org/), we failed to anchor any interactions between these proteins and the classical myogenic genes MyoG and MyoD (Supplement 3). But chi_circ_0008367 The parental gene EPDR1 is highly expressed in various tissues of goats, especially in skeletal muscle (Figure 1B), and is significantly elevated on day 3 of MuSCs differentiation in goats (Figure 1C).

Meanwhile, chi_circ_0008367 continually upregulated in LD muscles from E45 to B150 detected using RNA-seq and confirmed by RT-qPCR as well (Figure 1D; Supplement 2). Given that chi_circ_0008367 arises from the back-splicing of goat EPDR1 exons 2 and 3 (402 nt, Figure 1E; Supplement 4), we named it circEPDR1 (Figure 1E).

Moreover, we treated RNAs with RNase R to explore the stability of circEPDR1. As expected, the abundance of circEPDR1 was partially decreased by RNase R treatment (R+), compared with the control (R−). In contrast, the linear transcripts EPDR1 and GAPDH were completely digested by RNase R (Figure 1F). Additionally, we reverse-transcribed the total RNA using Random, Oligo dT primers (cDNA-R vs cDNA-O) separately and a common primer (cDNA). PCR results indicated that GAPDH mRNA was successfully amplified in cDNA and gDNA. In contrast, circEPDR1 only emerged using cDNA templates reverse-transcribed by primers containing the Random (cDNA-R and cDNA), instead of cDNA-O and gDNA, further confirming the circular nature of circEPDR1 (Figure 1G). Moreover, circEPDR1 was ubiquitously expressed in goat skeletal muscles and internal organs, with the highest levels in the LD muscle, followed by PM, ST, and GA (Figure 1H). Furthermore, during the proliferation phase (G1, G2, and D0), circEPDR1 steadily increased; then, it peaked on D3 and thereafter continued to decrease (Figure 1I), which roughly coincided with the profile of EPDR1 mRNA (Figures 1B, 1C). These results suggest that circEPDR1 likely plays a critical role in muscle development.

### CircEPDR1 inhibits proliferation but promotes myogenic differentiation of goat MuSCs

**Figure 2:**
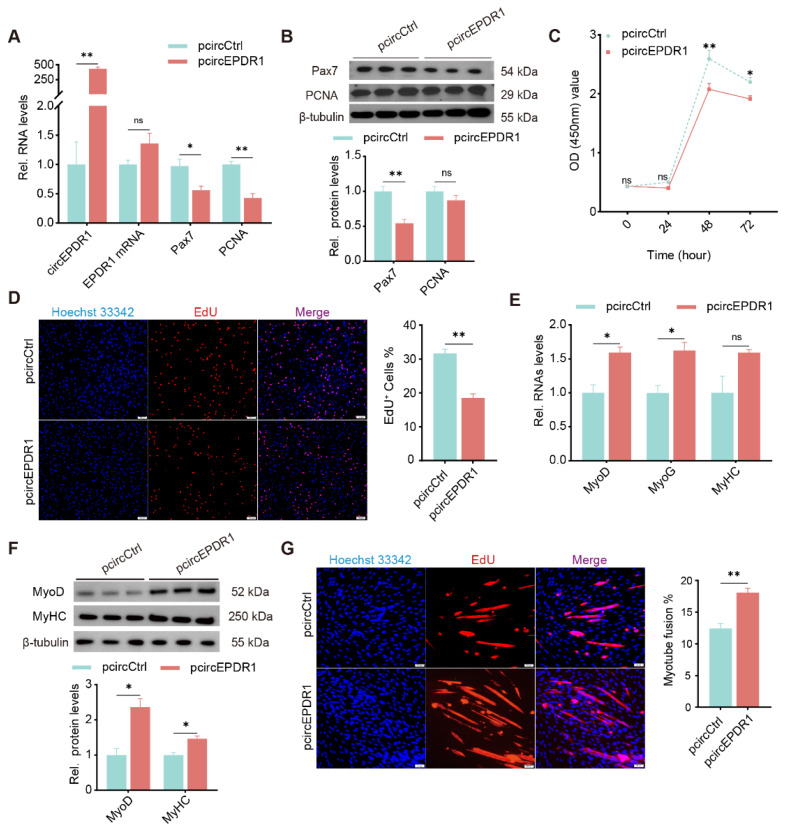
CircEPDR1 affects proliferation and myogenic differentiation of goat MuSCs. (A) Levels of circEPDR1, EPDR1 mRNA, Pax7 mRNA, and PCNA mRNA in proliferating MuSCs transfected with pCD5-circEPDR1(pcircEPDR1) or control (pcircCtrl). (B) Proteins of Pax7 and PCNA detected by WB in proliferating MuSCs. (C) Cell number measured by CCK-8 in proliferating MuSCs. (D) EdU positive cells (EdU^+^) affected by pcircEPDR1 or pcircCtrl. Cells in the s-phase were stained with EdU (red), and their nuclei were stained with Hoechst 33342 (blue) (magnification, ×100 μm). ImageJ V1.54 was used to count the proportion of EdU^+^ (EdU^+^/total number of cells). (E) MyoD, MyoG, and MyHC levels detected by RT-qPCR in differentiating cells treated with pCD5-circEPDR1 or control. (F) Proteins of MyoD and MyHC detected by WB in differentiating cells. (G) Myotubes immunofluorescence stained with antibody against MyHC in cells treated with pcircEPDR1 or pcircCtrl (magnification, ×50 μm), ImageJ V1.54 was employed to count myotube fusion rate (Number of multi-nucleated myotube nuclei/Total number of nuclei). Values are mean±SEM for at least three biological replicates, * p<0.05, ** p<0.01, ^ns^ p>0.05. MuSC, muscle satellite cell; RT-qPCR, real-time quantitative polymerase chain reaction; WB, Western blotting; SEM, standard error of the mean; NS, not significant.

To investigate the function of circEPDR1 in myogenesis, its overexpression vectors (pcircEPDR1) were constructed and transfected to cells. Compared with cells treated with the control (pcircCtrl), pcircEPDR1 treatment successfully upregulated circEPDR1 by ~400 fold change in proliferating MuSCs (p<0.01), with slight elevation of EPDR1 mRNA (p>0.05, Figure 2A). Meanwhile, transcripts of Pax7 and PCNA were dramatically downregulated by pcircEPDR1 (p<0.05 or p<0.01, Figure 2A), accordingly, their proteins were decreased (Figure 2B). Moreover, we employed CCK-8 and EdU to assay cell numbers. As expected, ectopic circEPDR1 significantly inhibited the optical density values, especially at 48 h and 72 h (Figure 2C), indicating a decrease in viable cells. Additionally, proliferating cells were stained, and the results showed that EdU-positive cells were dramatically declined by ectopic circEPDR1 (p<0.01, Figure 2D). These imply that crcEPDR1 retards the proliferation of goat MuSCs.

Furthermore, we transfected pcircEPDR1 into cells on the 2nd day of myogenic differentiation. The RT-qPCR results demonstrated that circEPDR1 significantly up-regulated the expression of differentiated genes MyoD, MyoG, and MyHC by ~1.5 fold (Figure 2E), though levels of MyHC were insignificantly changed (p>0.05). Additionally, we detected and found proteins of MyoD, MyHC also elevated by pcircEPDR1 (p<0.05, Figure 2F). Coincidingly, cell fusion rates presented as MyHC-staining were greatly promoted by ectopic circEPDR1 compared with controls (p<0.01, Figure 2G). These findings suggest that circEPDR1 promotes the myogenic differentiation of goat MuSCs.

### CircEPDR1 interacts with miR-345-3p

**Figure 3:**
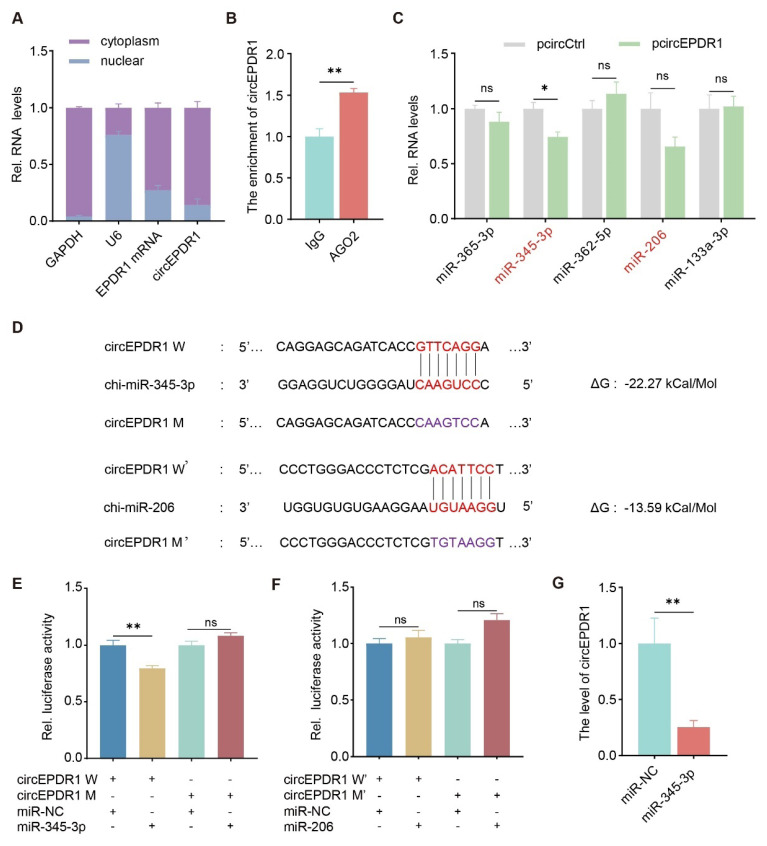
Interplay between circEPDR1 and miR-345-3p. (A) The subcellular localization of circEPDR1. GAPDH mRNA, U6, and EPDR1 mRNA work as the control. (B) CircEPDR1 enriched by AGO2 protein performed in RIP-qPCR assay. (C) miR-345-3p level detected by RT-qPCR in cells overexpressed circEPDR1. (D) Prediction of circEPDR1 binding with miR-345-3p. Red denoted the miR-345-3p or miR-206 seed sequence pairing with circEPDR1; purple represented the mutant sequence of cir-cEPDR1; ΔG values were obtained from miRanda. (E) The relative luciferase activity quantified in cells co-transfected with miR-345-3p mimics and wild-type or mutant cir-cEPDR1 vector (circEPDR1-W or circEPDR1-M). (F) Luciferase activity evaluated in cells treated by miR-206 mimics combined with wild-type or mutant circEPDR (cir-cEPDR1-W’ or circEPDR1-M’). (G) CircEPDR1 level detected in cells overexpressed miR-345-3p using RT-qPCR. Values are mean±SEM for three biological replicates, * p<0.05, ** p<0.01, ^ns^ p>0.05. GAPDH, glyceraldehyde-3-phosphate dehydrogenase; IgG, immunoglobulin G; AGO2, Argonaute2; miR-NC, negative control miRNA; RIP, RNA immunoprecipitation; RT-qPCR, real-time quantitative polymerase chain reaction; SEM, standard error of the mean; NS, not significant.

To investigate the underpinning mechanism of circEPDR1 in myogenesis, we first determined its subcellular localizations in cells. Using cDNA reversely transcribed from nuclear and cytoplasmic RNA separately, we quantified and found that circEPDR1 was predominantly distributed in the cytoplasm (~85%, Figure 3A). Next to explore whether circEPDR1 potentially interacts with miRNAs, we first employed an AGO2-RIP experiment combined with RT-qPCR. We found that circEPDR1 was significantly enriched by the AGO2 antibody, compared with the negative control IgG (Figure 3B).

Furthermore, we screened for miRNAs that may bind to circEPDR1. A total of 17 miRNAs were obtained (Supplement 5), among which we selected 5 miRNAs and found that the hybridization energy between miR-345-3p and circEPDR1 was the lowest (-22.27 kCal/Mol, Supplement 6). Moreover, miR-345-3p was the only one significantly downregulated by circEPDR1 (p<0.05, Figure 3C). To confirm the base-pairing between circEPDR1 and miR-345-3p, we precisely anchored their complementary sequences and constructed mutated circEPDR1 vectors (circEPDR1-M) to perform the dual-luciferase reporter assays, with miR-206 as a control (Figure 3D). The results revealed that in cells transfected with circEPDR1-W, the addition of miR-345-3p significantly reduced dual-luciferase activity compared to the control (negative control miRNA [miR-NC]) (p<0.01). In contrast, this inhibition of miR-345-3p was abolished once the complimentary sequence was mutated in circEPDR1 (circEPDR1-M) (p>0.05, Figure 3E). Moreover, the dual-luciferase activity of neither the wild nor mutant type of circEPDR1 (circEPDR1-W’ and circEPDR1-M’) was changed by the presence of miR-206 (p>0.05, Figure 3F). Furthermore, introducing miR-345-3p in cells dramatically decreased circEPDR1 levels by ~75% (p<0.01, Figure 3G). These findings robustly indicate that circEPDR1 interplays with miR-345-3p.

### miR-345-3p promotes proliferation and inhibits myogenic differentiation of goat MuSCs

**Figure 4:**
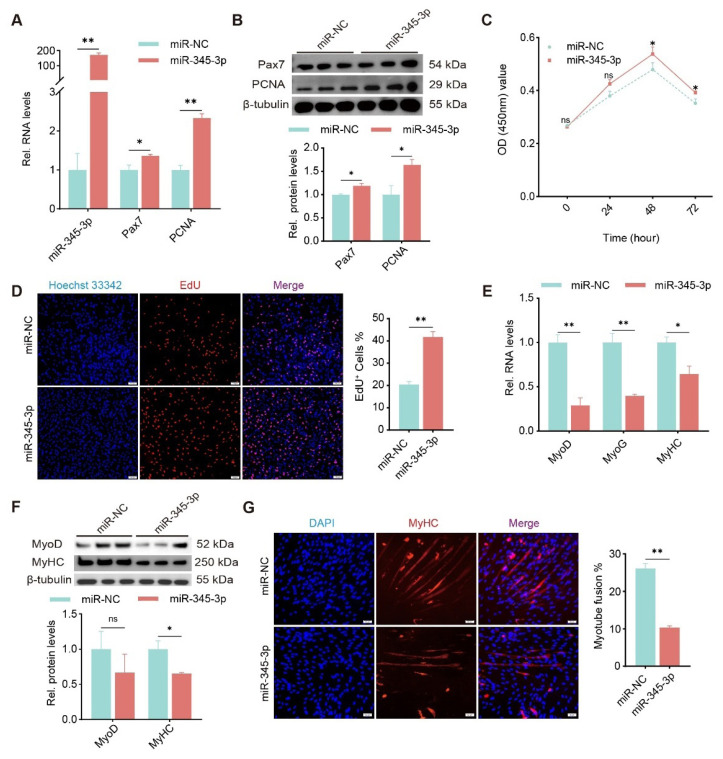
Functions of miR-345-3p in proliferation and myogenic differentiation of goat MuSCs. (A) Levels of miR-345-3p, Pax7, and PCNA transcripts detected in proliferating MuSCs transfected with miR-345-3p mimics (miR-345-3p) or control (miR-NC). (B) Proteins of Pax7 and PCNA detected by WB in differentiating MuSCs. (C) Cell number measured by CCK-8 assays performed in proliferating MuSCs. (D) EdU^+^ affected by miR-345-3p mimics and miR-NC. The number of cells in the s-phase was stained with EdU (red), and the nucleus was stained with Hoechst 33342 (blue) (magnification, ×100 μm). ImageJ V1.54 was used to count the proportion of EdU^+^ (EdU^+^/total number of cells). (E) Levels of MyoD, MyoG, and MyHC transcripts detected in differentiating MuSCs treated with miR-345-3p mimics or control. (F) Proteins of MyoD and MyHC detected in differentiating MuSCs. (G) Myotubes immunofluorescence stained with antibodies against MyHC. Myotubes were stained with MyHC (red), and the nuclei were stained with DAPI (blue) (magnification, ×50 μm). ImageJ V1.54 was employed to count the myotube fusion rate (Number of multinucleated myotube nuclei/Total number of nuclei). Values are mean±SEM for at least three biological replicates, * p<0.05, ** p<0.01, ^ns^ p>0.05. OD, optical density; miR-NC, negative control miRNA; DAPI, 4′,6-diamidino-2-phenylindole; MuSC, muscle satellite cell; SEM, standard error of the mean; NS, not significant.

To investigate the function of miR-345-3p in myogenesis, we transfected miR-345-3p mimics or control (miR-NC) into goat cells. The successful elevation of miR-345-3p significantly upregulated mRNA and protein of Pax7 and PCNA (p<0.05 or p<0.01, Figures 4A, 4B). Moreover, viable cells quantified using CCK-8 assays were also increased by ectopic miR-345-3p, especially at 48 h and 72 h (Figure 4C), and the percentage of EdU^+^ cells greatly promoted by miR-345-3p, compared with the control (p<0.01, Figure 4D).

In addition, miR-345-3p was transfected into differentiating MuSCs. The RT-qPCR results revealed that overexpression of miR-345-3p significantly reduced MyoD, MyoG, MyHC transcripts (p<0.05 or p<0.01, Figure 4E). Correspondingly, we detected and found proteins of MyoD and MyHC were downregulated (Figure 4F). Immunofluorescent staining for MyHC demonstrated that, compared with the myotube fusion rate of the control group (miR-NC, ~25%), miR-345 3p mimics significantly reduced cell fusion rate to ~10% (p<0.01, Figure 4G). These findings suggest that miR-345-3p promotes proliferation and inhibits differentiation of goat MuSCs, which is in reverse to the function of circEPDR1 mentioned above.

### CircEPDR1 regulates MuSCs proliferation and differentiation via miR-345-3p

**Figure 5:**
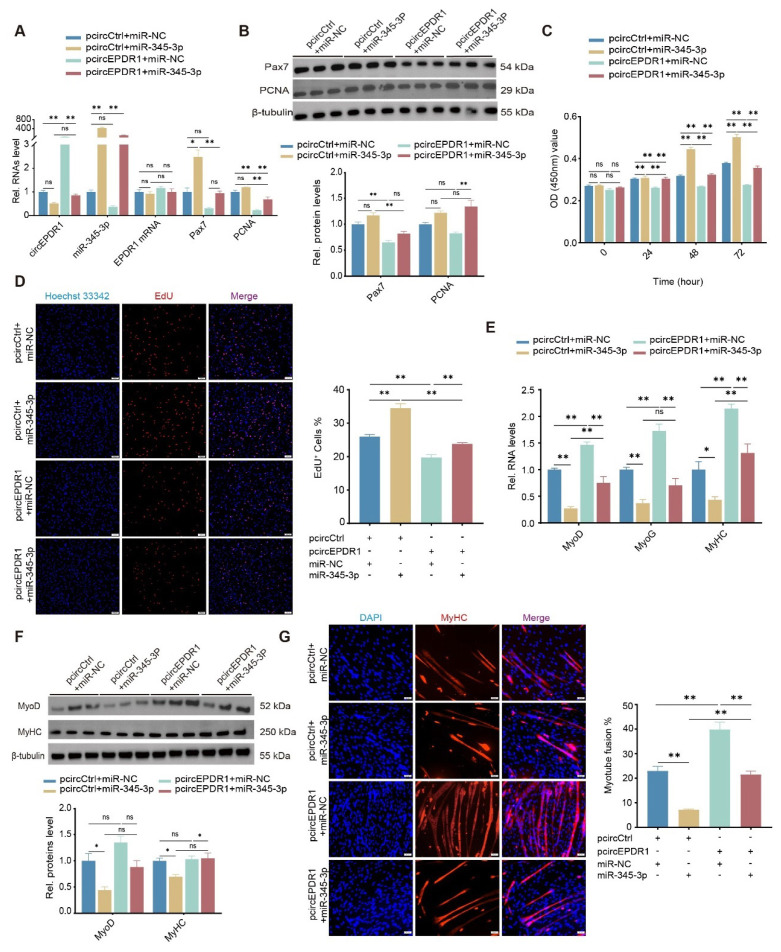
Effects of circEPDR1/miR-345-3p on proliferation and differentiation of goat MuSCs. (A) Levels of circEPDR1, miR-345-3p, EPDR1 mRNA, Pax7, and PCNA transcripts detected in proliferating MuSCs co-transfected pCD5-circEPDR1 with miR-345-3p mimics or control. (B) Proteins of Pax7 and PCNA detected by WB in differentiating MuSCs. (C) Cell number measured by CCK-8 performed in proliferating MuSCs. (D) EdU^+^ affected pCD5-circEPDR1 combined with miR-345-3p mimics. The number of cells in the s-phase was stained with EdU (red), and the nuclei were stained with Hoechst 33342 (blue) (magnification, ×100 μm). ImageJ V1.54 was used to count the proportion of EdU^+^ (EdU^+^/total number of cells). (E) MyoD, MyoG and MyHC levels detected by RT-qPCR in differentiating MuSCs treated with co-transfected pCD5-circEPDR1 with miR-345-3p mimics or control. (F) Proteins of MyoD and MyHC detected by WB in differentiating MuSCs. (G) Myotubes immunofluorescence stained with MyHC antibody. Cells were treated pCD5-circEPDR1 combined with miR-345-3p mimics or control, and myotubes were stained with antibodies against MyHC (red) and the nuclei with DAPI (blue) (magnification, ×50 μm). ImageJ V1.54 was employed to count the myotube fusion rate (Number of multinucleated myotube nuclei/Total number of nuclei). Values are mean±SEM for at least three biological replicates, * p<0.05, ** p<0.01, ^ns^ p>0.05. miR-NC, negative control miRNA; OD, optical density; DAPI, 4′,6-diamidino-2-phenylindole;e MuSC, muscle satellite cell; SEM, standard error of the mean; NS, not significant.

To further elucidate and confirm whether circEPDR1 functions on proliferation and differentiation of goat MuSCs through miR-345-3p, we performed transient co-transfection of pcircEPDR1 and miR-345-3p mimics into cells. As shown in Figure 5A, compared with the control (cells co-transfected pcircCtrl and miR-NC), the presence of miR-345-3p successfully downregulated circEPDR1 (cells co-transfected pcircCtrl and miR-345-3p mimics); inversely, ectopic circEPDR1 decreased miR-345-3p (cells co-transfected pcircEPDR1 and miR-NC). Nevertheless, levels of miR-345-3p and circEPDR1 in cells simultaneously transfected pcircEPDR1 and miR-345-3p mimics were insignificantly different from the control (p>0.05). And levels of EPDR1 mRNA failed to be altered by any of the treatments (p>0.05). These imply that circEPDR1 and miR-345-3p negatively regulate each other, which is irrelevant to the linear transcripts of the EDPR1 gene.

Compared with the control, miR-345-3p mimics up-regulated the mRNAs and proteins of Pax7 and PCNA when co-transfected with pcircCtrl, while oppositely, pcircEPDR1 downregulated Pax7 and PCNA in cells co-transfected with miR-NC (Figures 5A, 5B). Intriguingly, these effects were neutralized when circEPDR1 and miR-345-3p appeared simultaneously (Figures 5A, 5B). Furthermore, EdU and CCK-8 assay showed that adding miR-345-3p rescued the inhibitory effect of circEPDR1 on the number of viable and newly formed cells (p<0.01, Figures 5C, 5D).

During differentiation of goat MuSCs, cells co-transfected pcircCtrl and miR-345-3p mimics inhibited mRNA and protein of myogenic gene MyoD, MyoG, and MyHC. On the contrary, pcircEPDR1 combined with miR-NC treatment promoted these genes (Figures 5E, 5F). Intriguingly, these solely inhibiting or promoting effects were alleviated when co-transfected pcircEPDR1 with miR-345-3p (Figures 5E, 5F). Immunofluorescence results showed that miR-345-3p significantly restored circEPDR1’s promotion of myotube formation (p<0.01, Figure 5G). These results suggest that circEPDR1 inhibits the proliferation and promotes differentiation of goat MuSCs by targeting miR-345-3p.

### Akirin1 is regulated by circEPDR1/miR-345-3p

**Figure 6:**
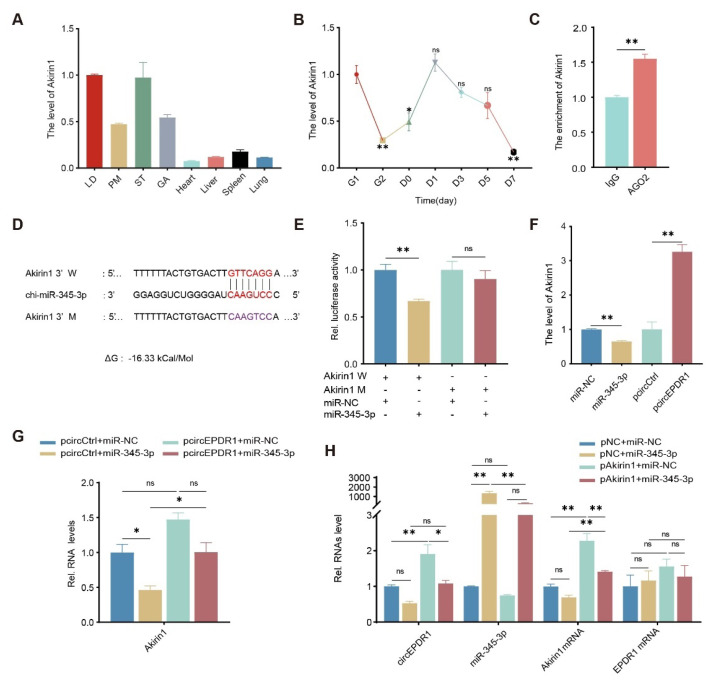
CircEPDR1/miR-345-3p axis regulates the expression of Akirin1. (A) The level of circEPDR1 detected by RT-qPCR in goat tissues. (B) Akirin1 levels detected by RT-qPCR in the proliferation and differentiation of goats MuSCs. (C) Akirin1 enriched by AGO2 performed in RIP assay. (D) Prediction of miR-345-3p binding with Akirin1. Red denoted the miR-345-3p seed sequence pairing with Akirin1; Purple represented the mutant sequence, and ΔG values were obtained from miRanda. (E) The relative luciferase activity quantified in cells co-transfected with miR-345-3p mimics and wild-type or mutant Akirin1 vector (Akirin1 W or Akirin1 M). (F) Akirin1 level detected by RT-qPCR in cells overexpressed miR-345-3p and circEPDR1. (G) Levels of Akirin1 mRNA transcript detected in proliferating MuSCs co-transfected pCD5-circEPDR1 with miR-345-3p mimics or control. pcircCtrl+miR-345-3p, pcir-cEPDR1+miR-NC, pcircEPDR1+miR-345-3p represented the treatment, and pcircCtrl+miR-NC represented control group. (H) Levels of circEPDR1, miR-345-3p, Akirin1 mRNA, and EPDR1 mRNA transcripts detected in proliferating MuSCs co-transfected PEGFP-Akirin1 with miR-345-3P mimics or control. pNC+miR-345-3P, pEGFP-Akirin1+ miR-NC, pEGFP-Akirin1+miR-345-3P represented the treatment, and PNC+miR-NC represented control group. Values are mean±SEM for three biological replicates, * p<0.05, ** p<0.01, ^ns^ p>0.05. LD, longissimus dorsi muscle; PM, psoas major muscle; ST, semitendinosus muscle; GA, gastrocnemius muscle; IgG, immunoglobulin G; AGO2, Argonaute2; miR-NC, negative control miRNA; pNC, negative control plasmid; RT-qPCR, real-time quantitative polymerase chain reaction; MuSC, muscle satellite cell.

To delve into the downstream target gene involved in circEPDR1/miR-345-3p, using resources from online websites and TargetScan databases, we first successfully predicted that 20 genes were potentially targeted by miR-345-3p (Supplement 5). Among these, Akirin1 has been unveiled to play a crucial myogenic role in skeletal muscle development and growth [21]. We detected a particularly high abundance of Akirin1 in skeletal muscles, including LD, PM, ST, and GA (Figure 6A). Additionally, we observed its decline during the myogenic differentiation of MuSCs (Figure 6B). Subsequently, we utilized RIP experiments and found that compared to the negative control IgG, AGO2 antibodies significantly enriched Akirin1 transcripts (p<0.01, Figure 6C). To confirm base-pairing between 3′ UTR of Akirin1 transcripts (Gene ID: 102170575), we constructed an overexpression vector for Akirin1 (pEGFP-Akirin1) and dual-luciferase reporter vectors containing both wild-type and mutant binding sites (Akirin1-W and Akirin1-M) (Figure 6C). We observed that compared with the control (miR-NC), miR-345-3p mimics significantly inhibited the luciferase activity of the Akirin1-W reporter vector (Akirin1-W, p<0.01). In contrast, this inhibition was abolished when the binding site was mutated (Akirin1-M, p>0.05) (Figure 6E). These suggest that the complementary sequence between miR-345-3p and Akirin1 is indispensable for their interaction.

To further uncover the intricate relationships among circEPDR1, Akirin1, and miR-345-3p, we quantified their levels in cells treated variously. The results revealed that miR-345-3p mimics significantly inhibited Akirin1 expression (p<0.01). On the contrary, overexpression of circEPDR1 up-regulated Akirin1 transcripts (p<0.01) (Figure 6F). Moreover, we transiently cotransfected cells and quantified Akirin1 mRNA. Compared to the control group (pcircCtrl+miR-NC), ectopic circEPDR1 or miR-345-3p alone increased or decreased Akirin1, coinciding with the results in cells that transfected them separately. The promotion of circEPDR1 on Akirin1 was neutralized by miR-345-3p in cells when they appeared simultaneously (Figure 6G).

Furthermore, we constructed overexpression vectors of Akirin1 (pAkirin1) and cotransfected it with miR-345-3p in cells. pAkirin1 treatment significantly elevated Akirin1 levels, and the addition of miR-345-3p successfully downregulated it regardless of overexpression Akirin1 or not. Similarly, circEPDR1 was almost halved by miR-345-3p but doubled by ectopic Akirin1 (p<0.01), while co-appearance of them neutralized these effects (Figure 6H). Reversely, compared with the cells treated with miR-345-3p mimics and negative control plasmid (pNC), miR-345-3p was consumed by extra Akirin1 (pAkirin1+miR-345-3p mimics). Importantly, none of the treatments affected the mRNA abundance of EPDR1 (p>0.05) (Figure 6H). These suggest that circEPDR1 and Akirin1 positively regulate each other and negatively associate with miR-345-3p, confirming that Akirin1 is a downstream gene of circEPDR1/miR-345-3p.

### miR-345-3p regulates proliferation and differentiation of goat MuSCs by targeting Akirin1

**Figure 7:**
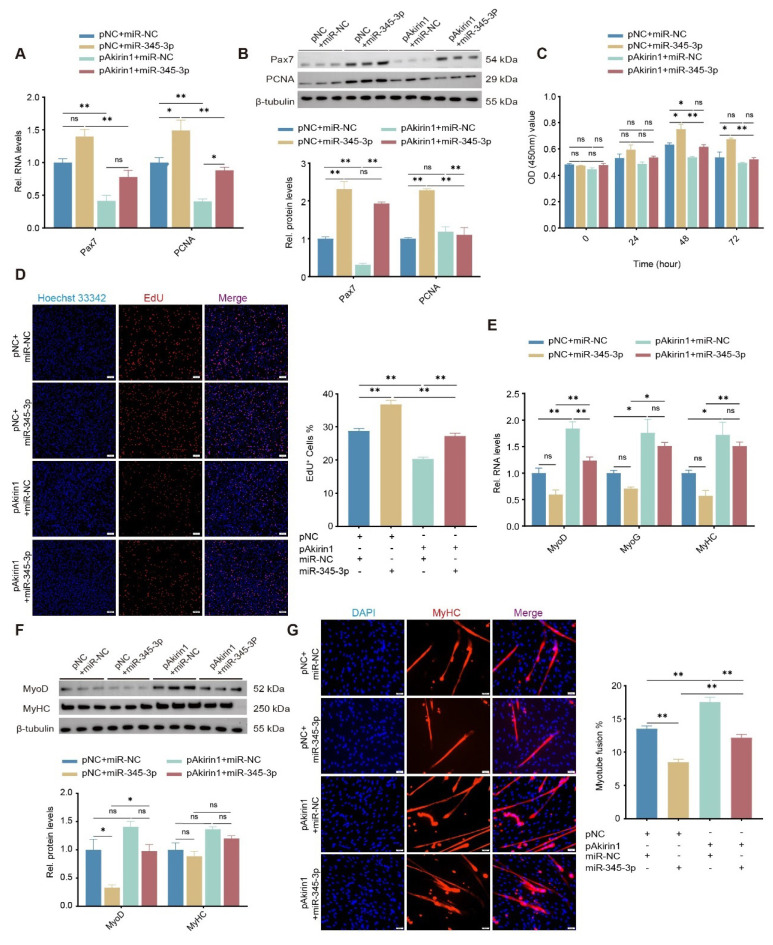
Regulation of Akirin1 on the proliferation and differentiation of goat MuSCs affected by miR-345-3p. (A) Pax7 and PCNA transcripts detected in proliferating MuSCs co-transfected pAkirin1 with miR-345-3p mimics or control. pNC and miR-NC represented the control. (B) Proteins of Pax7 and PCNA detected by WB in proliferating MuSCs. (C) Cell number measured by CCK-8 assays performed in proliferating MuSCs at four stages. (D) EdU^+^ affected by Akirin1 and miR-345-3p. The cell in the s-Phase was stained with EdU (red), and the nucleus was stained with Hoechst 33342 (blue) (magnification, ×100 μm). ImageJ V1.54 was used to count the proportion of EdU^+^ (EdU^+^/total number of cells). (E) MyoD, MyoG and MyHC mRNA detected by RT-qPCR in differentiating MuSCs co-transfected with pAkirin1 and miR-345-3p mim-ics or control. (F) Proteins of MyoD and MyHC detected by WB in differentiating MuSCs. (G) Myotubes immunofluorescence stained with MyHC antibody. Cells were co-transfected pAkirin1 with miR-345-3p mimics or control, and myotubes were stained with antibodies against MyHC (red) and the nucleus with DAPI (blue) (magnification, ×50 μm). ImageJ V1.54 was employed to count the myotube fusion rate (Number of multinucleated myotube nuclei/Total number of nuclei). Values are mean±SEM for three biological replicates, * p<0.05, ** p<0.01, ^ns^ p>0.05. pNC, negative control plasmid; miR-NC, negative control miRNA; MuSC, muscle satellite cell; WB, Western blotting; RT-qPCR, real-time quantitative polymerase chain reaction; DAPI, 4′,6-diamidino-2-phenylindole; SEM, standard error of the mean; NS, not significant.

To further elaborate on the interaction between miR-345-3p and Akirin1 in regulating the proliferation and differentiation of goat MuSCs, we transiently co-transfected pAkirin1 and miR-345-3p mimics into cells. Compared to the control group, when pAkirin1 was co-transfected with miR-NC, the mRNA and protein levels of both Pax7 and PCNA were inhibited in proliferating cells (Figures 7A, 7B). Conversely, they were increased by miR-345-3p mimics alongside pNC (Figures 7A, 7B). Notably, the inhibition of Akirin1 on Pax7 and PCNA expression was rescued when miR-345-3p coexisted in the cells (Figures 7A, 7B). Furthermore, results from EdU and CCK-8 experiment showed that adding Akirin1 restrained newly formed cells and viable cells, miR-345-3p efficiently released this hold-up (p*<*0.01, Figures 7C, 7D).

Similarly, ectopic Akirin1 significantly up-regulated the mRNA of differentiation markers MyoD, MyoG, and MyHC (p<0.05, Figure 7E), and correspondingly, the protein of both MyoD and MyHC were elevated (Figure 7F). Notably, these blooms were mitigated when miR-345-3p was co-existed. Immunofluorescence results further confirmed that miR-345-3p dramatically retained the promoting effect of Akirin1 on myotube formation (p<0.01, Figure 7G). These suggest that similar to circEPDR1, Akirin1 also inhibits proliferation but promotes myogenic differentiation of goat MuSCs, which is efficiently retarded by miR-345-3p.

## DISCUSSION

CircRNA is essential for skeletal muscle development and is strongly associated with meat yield and quality [22], and it is abundantly expressed in skeletal muscles and play pivotal roles in regulating gene expression and protein function [23]. Some circRNAs are able to precisely regulate the proliferation and differentiation process of muscle-forming cells at specific stages, which in turn affects muscle growth and development [24]. However, continuous inhibition of MuSCs proliferation may damage satellite cells and ultimately have an adverse effect on the long-term growth potential of muscle [25]. Here, we analyzed circRNA sequencing data from the LD muscle of goats at key developmental stages and identified nine differentially expressed circRNAs. Intriguingly, their associated proteins do not interact with classical muscle-forming genes like MyoG, indicating novel regulatory pathways. The EPDR1 gene, highly abundant in human and mouse muscle tissues [26], has been implicated in various key cellular processes such as cell proliferation, myofibroblast contraction, and osteoblast differentiation [27–29]. Subsequently we identified circEPDR1 that originated from the circularization of exons 2 and 3 of the EPDR1 gene. EPDR1 and circEPDR1 were significantly enriched in skeletal muscles. During the proliferative phase of MuSCs, the levels of these two substances first increased and then decreased. This trend suggested that continuous inhibition of MuSCs proliferation would weaken the strength and regenerative capacity of skeletal muscle. During the D1 to D3 phase of myogenic differentiation, their levels increased, indicating that regulating their levels during this phase might promote skeletal muscle regeneration and development. These high expressions and dynamic changes at different stages imply that they might be in response to the diverse requirements of muscle development. In addition, circEPDR1 exhibited relatively high resistance to RNase R. Its circular nature was confirmed by PCR identification and Sanger sequencing. CircEPDR1 inhibits the proliferation of goat MuSCs but promotes their differentiation, a phenomenon that suggests that circEPDR1 is involved in skeletal muscle development and regulates its regenerative process.

Sponging miRNA is the most common biological function unveiled for cytosol circRNAs [30]. Previous research exposed that circFgfr2/miR-133/Map3k20 in pigs [31] and circARID1A/miR-6368 in mice [32] inhibit myoblast proliferation and promote its myogenic differentiation. While circCPE/miR-138/FOXC1 enhances bovine myoblast proliferation and inhibits differentiation [24]. Moreover, circSVIL promotes the proliferation and differentiation of chicken myoblasts by sponging miR-203 and consequently sequestering c-JUN and MEF2C, respectively [33]. We found that circEPDR1 was dominantly distributed in the cytoplasm and enriched by the AGO2 protein, a core member of miRNA machinery, demonstrating that circEPDR1 has the potential to act as a ceRNA In addition, miR-345-3p was identified as potentially targeting circEPDR1, the complementary sequences of the two were confirmed by dual luciferase reporter analysis. miR-345-3p promoted the proliferation of goat MuSCs while inhibiting their differentiation, which is in contrast to the function of circEPDR1 on goat MuSCs. miR-345-3p mitigated the effects of circEPDR1 on MuSCs proliferation and differentiation, when co-transfected, miR-345-3p and circEPDR1 mutually exerted negative regulation without influencing EPDR1 mRNA. Their interaction counteracted each other’s impacts on marker genes and proteins associated with MuSCs proliferation and differentiation, thereby modulating cell behavior. This negative regulatory relationship likely functions as a balancing mechanism to ensure the orderly conduct of cell proliferation and differentiation, indicating that circEPDR1 regulates MuSCs proliferation and differentiation via its binding to miR-345-3p.

We further predicted and validated Akirin1 as a target of miR-345-3p (Supplement 5). Akirin1 is a highly conserved and ubiquitously expressed muscle-promoting factor, functioning as a crucial downstream regulator of muscle inhibitory proteins [34] We found that Akirin1 is highly expressed in skeletal muscle and decreases during the proliferation of MuSCs, and then increases and decreases during the differentiation of MuSCs, which is similar to the expression pattern of circEPDR1 in tissues and cells. This expression pattern is similar to that of circEPDR1 in tissues and cells. This implies that Akirin1 can continuously inhibit MuSCs during the proliferative phase, and without impairing the function of MuSCs, it can promote differentiation at the early stage of differentiation, and then Akirin1 will continue to decline to maintain the homeostasis after reaching the normal muscle development and regeneration state. In addition, Akirin1 is enriched in AGO2 protein. Dual-luciferase reporter assays demonstrated that circEPDR1 shared miR-345-3p binding sites with Akirin1, and the binding energy is lower in circEPDR1/miR-345-3p (−22.27 kcal/mol) than miR-345-3p/Akirin1 (−16.33 kcal/mol), indicating it is more efficient for circEPDR1 to absorb miR-345-3p than Akirin1 transcripts, and this also confirmed that miR-345-3p interacts with Akirin1 and that Akirin1 is a downstream gene of miR-345-3p. Co-transfection experiments revealed that akirin1 overexpression inhibits cell proliferation and promotes differentiation, and this effect is affected by miR-345-3p, while miR-345-3p inhibits Akirin1 expression, circEPDR1 upregulates Akirin1 transcripts, and the regulatory effects of miR-345-3p and circEPDR1 cancel each other when they are present at the same time. These results robustly suggest that circEPDR1/miR-345-3p regulates the proliferation and differentiation of goat MuSCs by targeting Akirin1.

However, as a skeletal muscle enriched molecule, miR-345-3p/Forkhead box protein O1 likely affects goat intramuscular fat metabolism [35]. Additionally, RING1 and YY1 binding protein is also targeted by miR-345-3p in human trophoblast cells [36]. Therefore, except for Akirin1, other target genes possibly involved in circEPDR1/miR-345-3p in regulating proliferation and differentiation of goat MuSCs, and circEPDR1/miR-345-3p may also function in intramuscular fat.

In addition, certain circRNAs associated with m^6^A modification encode small peptides or proteins and play key roles in the complex process of skeletal muscle development [37,38]. Based on this, we discovered that circEPDR1 harbors three potential high-confidence sites for m^6^A modification and is likely to interact with enzymes crucial for m6A modification (Supplement 7A), particularly with Methyltransferase-like 3 and Wilms tumor 1 associated protein, both being key components of the methyltransferase complex. Furthermore, it may also interact with demethylase (ALKBH5) and methyl-reading protein (YTHDC1) (Supplement 7B). Moreover, circEPDR1 harbors a unique open reading frame spanning its cyclization site (Supplement 7C), a structure that strongly indicates the coding power for circRNAs [39,40]. Therefore, it would be interesting to experimentally validate the encoding capacity of circEPDR1, and reveal how m^6^A modifications dynamically regulate circEPDR1 translation in skeletal muscle development.

## CONCLUSION

**Figure 8:**
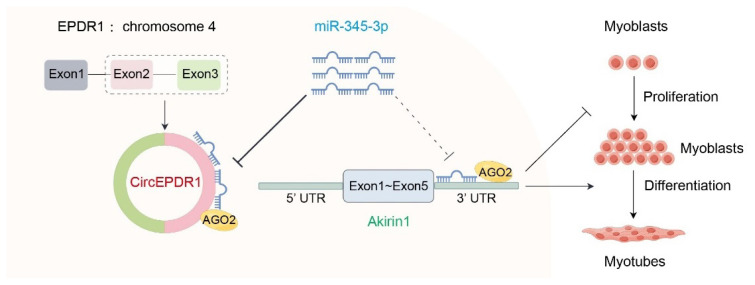
Schematic representation of circEPDR1/miR-345-3p/Akirin1 in proliferation and myogenic differentiation of goat MuSCs. circEPDR1 is formed by reverse splicing the second and third exons of the EPDR1 gene. By competitively binding to miR-345-3p, circEPDR1 sequesters the inhibitory effect of miR-345-3p on Akirin1 mRNA, thus inhibiting the proliferation of MuSCs and promoting their differentiation. MuSC, muscle satellite cell.

In summary, this study identified circEPDR1 as a critical player in goat skeletal muscle development. circEPDR1 was predominantly distributed within the cytoplasm and enriched by the AGO2 protein. By competitively binding miR-345-3p and targeting Akirin1, circEPDR1 reduced its suppressive effect of miR-345-3p on Akirin1 mRNA, thus inhibiting the proliferation and promoting the differentiation of goat MuSCs These discoveries offer a fresh perspective on the regulatory mechanisms of circRNAs in muscle development (Figure 8).

## SUPPLEMENTARY MATERIAL

**Supplement 1**. Primers for correlative experiment

**Supplement 2**. Information of 9 differentially expressed circRNAs screened in goat LD muscles at four developmental stages

**Supplement 3**. String-map for the connection of annotated genes with myogenic gene MyoD1 and MyoG.

**Supplement 4**. circ_0008367 produced by EPDR1

**Supplement 5**. The chi-circEPDR1-miRNA-mRNA Regulatory Network.

**Supplement 6**. The base-pairing site and hybridization energy between miRNAs and circEPDR1

**Supplement 7**. m^6^A modification and translation potential of cricEPDR1.
