# Supplementary material for: CircEPDR1 regulates proliferation and differentiation of goat skeletal muscle satellite cells through miR-345-3p/Akirin1 axis
Source: Anim Biosci. 2025 Mar 31;38(8):1605–21. doi: 10.5713/ab.24.0845 (PMC12229913; doi:10.5713/ab.24.0845)
Supplement: Supplementary file 1 [file ab-24-0845-Supplementary-1.pdf]

Supplement 1. Primers for correlative experiment

Gene name	Sequence 5'-3'	Tm (°C)	Size (bp)	Function	Gene ID
CircEPDR1	F: TATCCTGTCCAAGAAACCT<br>R: TAACAATCCTTGACGGTAT	59	402	FS	102175182
CircEPDR1	F: GAGAGGATGAGCGAAGAATGC<br>R: TGGTGGCTTGTTCGATCTGA	59.4	90	qPCR	102175182
CircEPDR1	F: AAAGACCCATCCGTGTTCAC<br>R: GGCTCTGTCAGGGTGATCTT	58.4	172	SEQ	102175182
EPDR1	F: CTTACGACGGGCTCAACCA<br>R GAGGAATGTCGAGAGGGTCC	60	183	qPCR	102175182
GAPDH	F: GCAAGTTCCACGGCACAG<br>R: GGTTCACGCCCATCACAA	59	249	qPCR	100860872
PCNA	F: GGAGAACTTGGAAATGGAAATA<br>R: TGTAGGAGACAGTGGAGTGGCT	61.3	156	qPCR	102172276
Pax7	F: AGGACGAAGCGGACAAGAA<br>R: TCCAGACGGTTCCCTTTGT	59.7	92	qPCR	102172220
MyoD	F: GTGCAAACGCAAGACGACTA<br>R: GCTGGTTTGGGTTGCTAGAC	60.7	128	qPCR	108637637
MyoG	F: GGACCCTACAGATGCCCACAA<br>R: TTGGTATGGTTTCATCTGGG	60.7	101	qPCR	100861395
MyHC	F: CCACATCTTCTCCATCTCTG<br>R: GGTTCCTCCTTCTTCTTCTC	61.3	171	qPCR	106502098
miR-345-3p	F: GCGCCCTGAACTAGGGGTC	60	——	qPCR	MIMAT0036164
Akirin1	F: AAAAGGACCAGCCCACCTTT<br>R: GGCCTTGTCCCATATCGTCG	60.7	184	qPCR	102170575

*: FS stands for primers used to amplify circEPDR1 full sequence.
