# Supplementary material for: CircEPDR1 regulates proliferation and differentiation of goat skeletal muscle satellite cells through miR-345-3p/Akirin1 axis
Source: Anim Biosci. 2025 Mar 31;38(8):1605–21. doi: 10.5713/ab.24.0845 (PMC12229913; doi:10.5713/ab.24.0845)
Supplement: Supplementary file 2 [file ab-24-0845-Supplementary-2.pdf]

Supplement 2. Information of 9 differentially expressed circRNAs screened in goat LD muscles at four developmental stages

CircRNA_ ID	E45_1 .tpm	E45_2 .tpm	E105_ 1.tpm	E105_ 2.tpm	B3_1. tpm	B3_2. tpm	B150_ 1.tpm	B150_ 2.tpm	mRN A_ID	Gene name	Gene type
chi_circ_0 014195	7082	5072	2228	2694	629	1120	158	214	10217 3553	FBN2	protein coding
chi_circ_0 034012	267	217	4357	5458	1405	2030	22	37	10219 1280	LOC1021 91280	protein coding
chi_circ_0 034149	1056	828	7271	6749	1254	1159	101	65	n/a	——	——
chi_circ_0 034148	5016	4295	16720	22819	1803	662	267	261	10650 3383	LOC1065 03383	ncRNA
chi_circ_0 008367	303	280	804	946	1978	2719	8579	8222	10217 5182	EPDR1	protein coding
chi_circ_0 008610	151	38	487	510	115	196	7	0	10218 3035	CACNA2 D1	protein coding
chi_circ_0 023846	120	83	379	432	1079	1028	313	158	10218 3078	HACD1	protein coding
chi_circ_0 003071	0	0	16	22	12	0	29	0	10217 2093	AP2B1	protein coding
chi_circ_0 000474	294	293	626	833	4200	5150	12818	19733	10219 1740	PPP2R3A	protein coding

*: The full names of genes are FBN2 (fibrillin 2), LOC102191280 (proline-rich receptor-like protein kinase PERK8), LOC106503383 (un characterized ncRNA), EPDR1 (ependymin related 1), CACNA2D1 (calcium voltage-gated channel auxiliary subunit alpha2delta 1), HA CD1 (3-hydroxyacyl-CoA dehydratase 1) AP2B1 (adaptor related protein complex 2 subunit beta 1), PPP2R3A (protein phosphatase 2 reg ulatory subunit B''alpha); E45: embryonic day 45; E105: embryonic day 105; B3: postnatal day 3; and B150: postnatal day 150.
