# Supplementary figures and images for: CircEPDR1 regulates proliferation and differentiation of goat skeletal muscle satellite cells through miR-345-3p/Akirin1 axis
Source: Anim Biosci. 2025 Mar 31;38(8):1605–21. doi: 10.5713/ab.24.0845 (PMC12229913; doi:10.5713/ab.24.0845)

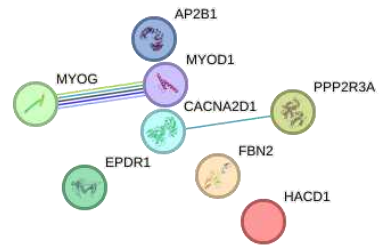

**Supplement 3.** String-map for the connection of annotated genes with myogenic gene MyoD1 and MyoG.

Supplement: Supplementary file 3 [file ab-24-0845-Supplementary-3.pdf]
