# Supplementary material for: CircEPDR1 regulates proliferation and differentiation of goat skeletal muscle satellite cells through miR-345-3p/Akirin1 axis
Source: Anim Biosci. 2025 Mar 31;38(8):1605–21. doi: 10.5713/ab.24.0845 (PMC12229913; doi:10.5713/ab.24.0845)
Supplement: Supplementary file 4 [file ab-24-0845-Supplementary-4.pdf]

Supplement 4. circ_0008367 produced by EPDR1

>chi_circ_0008367 NC_030811.1 70521838 70523336 - 1498 400

102175182; exon2: 70521839-70522031 exon3:70523128-70523336

ATTATTTGAATATATTTTGCTCTATAAGGATGGAGTGATGTTTCAGATCGAACAAGCCAC

CAAGCAGTGCTCCAAGATCACCCTGACAGAGCCCTGGGACCCTCTCGACATTCCTCAG

AACTCCACCTTTGAGGACCAATACTCCATAGGGGGGCCCCAGGAGCAGATCACCGTTC

AGGAATGGTCAGACAGAAAGTCAGCCAGATCATATGAAACTTGGATTGGTATATATACC

GTCAAGGATTGTTATCCTGTCCAAGAAACCTTCACCAAAAATTACAGTGTGATATTGTC

CACACGGTTTTTTGACATACAGCTGGGCATTAAAGACCCATCCGTGTTCACCCCACCAA

GCACGTGCCAGACAGCCCAACCAGAGAGGATGAGCGAAGAATGCTCCTG

>chi_circ_0008367_junction_seq

CCAGAGAGGATGAGCGAAGAATGCTCCTGATTATTTGAATATATTTTGCTCTATAAGG
