# Supplementary material for: CircEPDR1 regulates proliferation and differentiation of goat skeletal muscle satellite cells through miR-345-3p/Akirin1 axis
Source: Anim Biosci. 2025 Mar 31;38(8):1605–21. doi: 10.5713/ab.24.0845 (PMC12229913; doi:10.5713/ab.24.0845)
Supplement: Supplementary file 5 [file ab-24-0845-Supplementary-5.pdf]

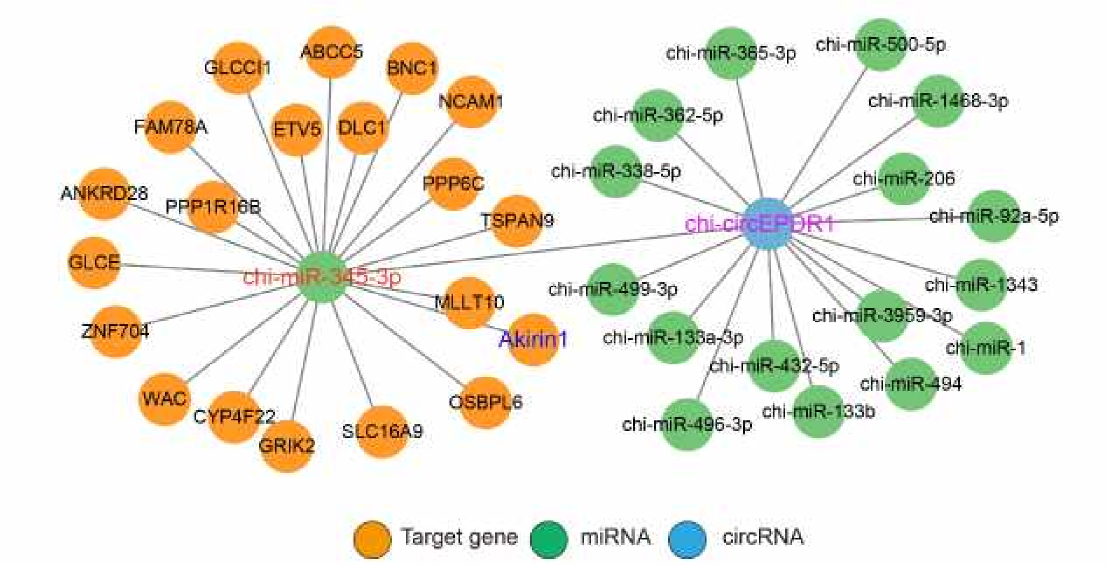

Supplement 5. The chi-circEPDR1-miRNA-mRNA Regulatory Network. Based on predictions from miRDB, miRwalk, miRmap and TargetScan databases, built the PPI network for the circRNA-miRNA-Target gene utilizing the Cytoscape software.
