# Supplementary material for: CircEPDR1 regulates proliferation and differentiation of goat skeletal muscle satellite cells through miR-345-3p/Akirin1 axis
Source: Anim Biosci. 2025 Mar 31;38(8):1605–21. doi: 10.5713/ab.24.0845 (PMC12229913; doi:10.5713/ab.24.0845)
Supplement: Supplementary file 6 [file ab-24-0845-Supplementary-6.pdf]

Supplement 6. The base-pairing site and hybridyzation energy between miRNAs and circEPDR1

RNA	Accession	Sequence	ΔG values (kCal/Mol)
Chi-miR-133a-3p	MIMAT0035948	3’ TGUCGACCAACUUCCCCUGGUUU 5’	-12.77
CircEPDR1		5’…GAACTCCACCTTTGAGGACCAAT…3’	
Chi-miR-206	MIMAT0036052	3’ UGGUGUGUGAAGGAAUGUAAGGU 5	-13.59
CircEPDR1		5’… CTGGGACCCTCTCGACATTCCT… 3’	
Chi-miR-365-3p	MIMAT0036177	3’ UAUUCCUAAAAAUCCCCGUAAU 5’	-11.87
CircEPDR1		5’ …TTTGACATACAGCTGGGCATTA… 3’	
Chi-miR-362-5p	MIMAT0036173	3’ UGAGUGUGGAUCCAAGGUUCCUAA 5’	-12.42
CircEPDR1①		5’…GAATATATTTTGCTCTATAAGGATG…3’	
Chi-miR-362-5p	MIMAT0036173	3’ UGAGUGUGGAUCCAAGGUUCCUAA 5’	-19.05
CircEPDR1②		5’ …TGGTATATATACCGTCAAGGATT…3’	
Chi-miR-345-3p	MIMAT0036164	3’ GGAGGUCUGGGGAUCAAGUCCC 5’	-22.27
CircEPDR1		5’ …GGAGCAGATCACCGTTCAGGA…3’	

*: The binding sequence was obtained based on miRanda analysis; Green sequence represents the complementary sequences between circEPDR1 and miRNAs; ①② means two base-pairing sites between Chi-miR-362-5p and CircEPDR1.
