# Supplementary material for: CircEPDR1 regulates proliferation and differentiation of goat skeletal muscle satellite cells through miR-345-3p/Akirin1 axis
Source: Anim Biosci. 2025 Mar 31;38(8):1605–21. doi: 10.5713/ab.24.0845 (PMC12229913; doi:10.5713/ab.24.0845)
Supplement: Supplementary file 7 [file ab-24-0845-Supplementary-7.pdf]

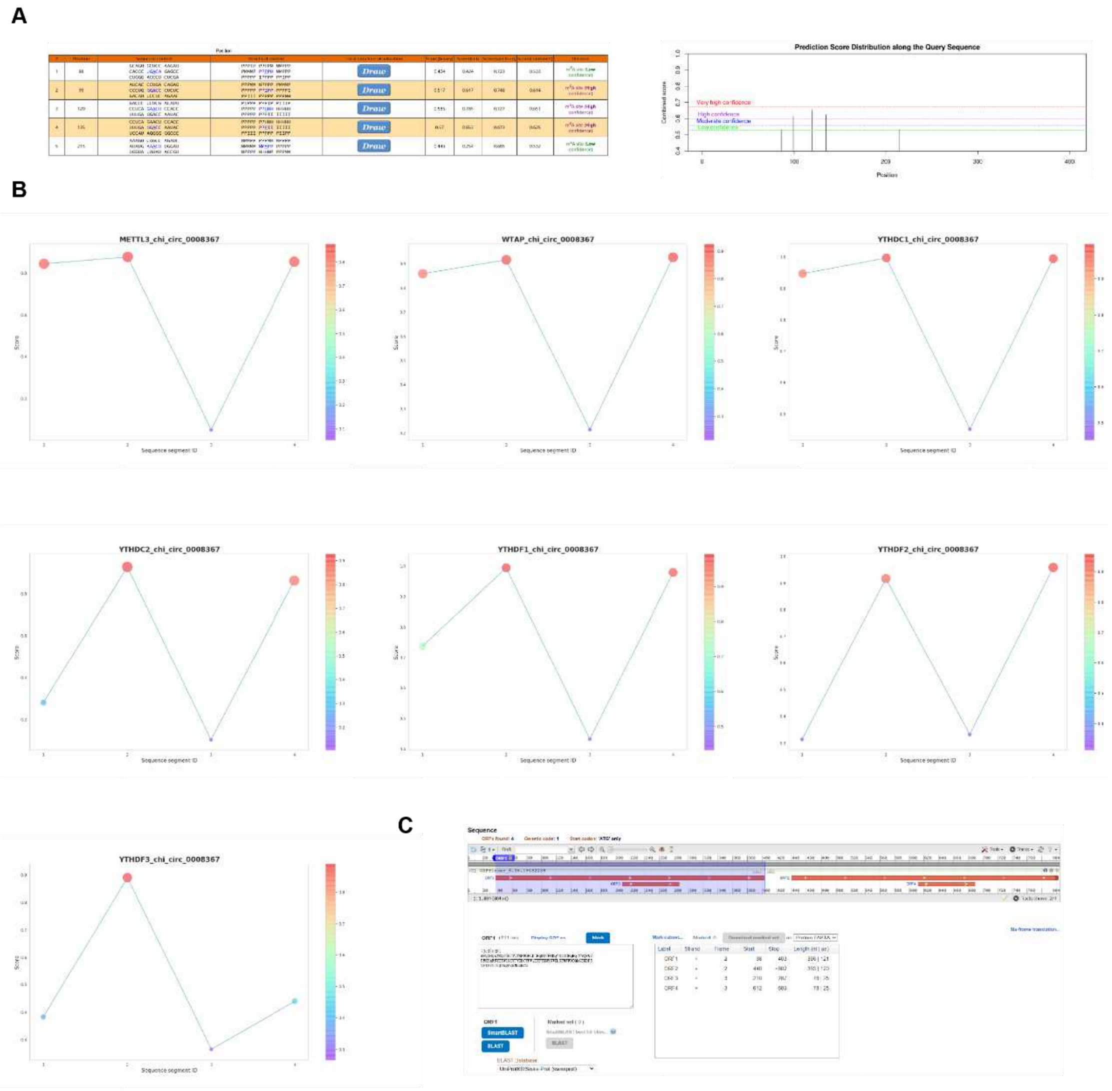

Supplement 7. m^{6}A modification and translation potential of cricEPDR1. **A** m^{6}A modification sites predicted on circEPDR1. **B** Potential interaction between m^{6}A modification-associated RNA-binding proteins and circEPDR1. **C** Open reading frames (ORF) predicted on circEPDR1.
